# Vertical distributions of soil microbial biomass carbon: a global dataset

**DOI:** 10.1016/j.dib.2020.106147

**Published:** 2020-08-08

**Authors:** Tingting Sun, Yugang Wang, Dafeng Hui, Xin Jing, Wenting Feng

**Affiliations:** a State Key Laboratory of Desert and Oasis Ecology, Xinjiang Institute of Ecology and Geography, Chinese Academy of Sciences, Urumqi, Xinjiang 830011, China; b University of Chinese Academy of Sciences, Beijing 100049, China; c National Fukang Station of Desert Ecosystem Ecology, Field Sciences Observation and Research Station, Chinese Academy of Sciences, Fukang, Xinjiang 831505, China; d Department of Biological Sciences, Tennessee State University, Nashville, TN 37209, USA; e Department of Earth and Environmental Sciences, KU Leuven, Celestijnenlaan 200E, 3001 Leuven, Belgium; f Institute of Agricultural Resources and Regional Planning, Chinese Academy of Agricultural Sciences, Beijing 100081, China

**Keywords:** deep soils, micorbial quotient, soil organic carbon, soil clay content, soil C/N ratio, soil profile

## Abstract

Soil microbial biomass carbon (SMBC) is important in regulating soil organic carbon (SOC) dynamics along soil profiles by mediating the decomposition and formation of SOC. The dataset (VDMBC) is about the vertical distributions of SOC, SMBC, and soil microbial quotient (SMQ = SMBC/SOC) and their relations to environmental factors across five continents. Data were collected from literature, with a total of 289 soil profiles and 1040 observations in different soil layers compiled. The associated environment data collectd include climate, ecosystem types, and edaphic factors. We developed this dataset by searching the Web of Sciene and the China National Knowledge Infrastructure from the year of 1970 to 2019. All the data in this dataset met two creteria: 1) there were at least three mineral soil layers along a soil profile, and 2) SMBC was measured using the fumigation extraction method. The data in tables and texts were obtained from literature directly, and the data in figures were extracted by using the GetData Graph digitizer software version 2.25. When climate and soil properties were not available from publications, we obtainted the data from the World Weather Information Service (https://worldweather.wmo.int/en/home.html) and SoilGrids at a spatial resolution of 250 meters (version 0.5.3, https://soilgrids.org).

The units of all the variables were converted to the standard international units or commonly used ones and the values were transformed correspondingly. For example, the value of soil organic matter (SOM) was converted to SOC by using the equation (SOC = SOM × 0.58).

This dataset can be used in predicting global SOC changes along soil profiles by using the multi-layer soil carbon models. It can also be used to analyse how soil microbial biomass changes with plant roots as well as the composition, structure, and functions of soil microbial communities along soil profiles at large spatial scales. This dataset offers opportunities to improve our prediction of SOC dynamics under global changes and to advance our understanding of the environmental controls.

Specifications Table

**Table:** 

Subject	Agricultural and Biological Science; environmental Science
Specific subject area	Soil carbon cycling, soil microorganisms
Type of data	txt.file, docx.file
How data were acquired	Systematic review of publications
Data format	Secondary data
Parameters for data collection	The keywords used to search relevant publications are microbial biomass, soil depth, soil profile, deep soil(s), subsoil(s), and vertical
Description of data collection	Data were collected from publications by searching the Web of Science and the China National Knowledge Infrastructure from the year of 1970 to 2019. The data about soil profiles reported in tables and text were obtained from literature directly, and the data in figures were extracted by using the GetData Graph digitizer software version 2.25. When the data of climate and soil properties other than microbial biomass are not available in publications, we obtained the data from the World Weather Information Service (https://worldweather.wmo.int/en/home.html) and SoilGrids at a spatial resolution of 250 meters (version 0.5.3, https://soilgrids.org) at the resolution of 250 meters according to the coordinates of soil profiles.
Data source location	Global
Data accessibility	The data can be retrieved from https://doi.org/10.5281/zenodo.3971022
Related research article	Tingting Sun, Yugang Wang, Dafeng Hui, Xin Jing, Wenting Feng. 2020. Soil properties rather than climate and ecosystem type control the vertical variations of soil organic carbon, microbial carbon and microbial quotient. Soil Biology & Biochemistry, https://doi.org/10.1016/j.soilbio.2020.107905

## 1 Value of the data

• Soil microbial biomass carbon is a key parameter in the classical and newly developed models of soil carbon dynamics, and this is the first dataset about the vertical distributions of soil microbial biomass carbon, organic carbon, and their ratio at the global scale.
• This dataset enables modelers to improve the prediction of vertical changes in soil organic carbon at large spatial scales. It also enables ecosystem ecologists to explore how soil microbial biomass changes with plant roots as well as the composition, structure, and functions of soil microbial communities along soil profiles.
• Together with the data of vertical distributions of soil organic carbon, plant roots, and other soil properties that are already publicly available, this dataset can be used in the multi-layer soil carbon models to simulate changes in soil organic carbon at different soil depths at large spatial scales.
• This dataset enables researchers to quantify how soil microbial biomass carbon and soil organic carbon change along soil profiles and to understand the underlying mechanisms. This will help improve the prediction of soil organic carbon dynamics under global changes in the terrestrial ecosystems.

## 2 Data description

**Fig. 1:**
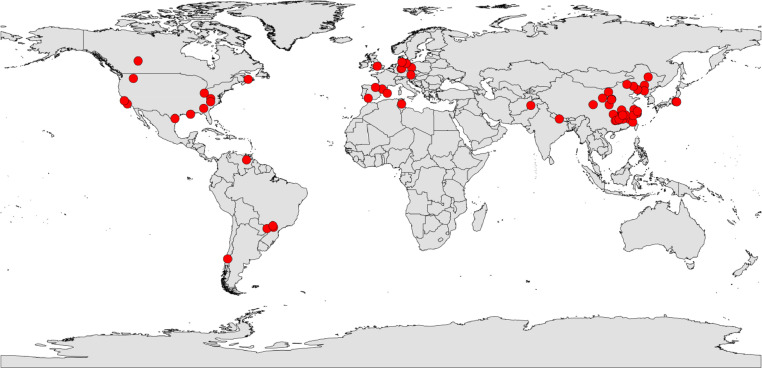
A global map of soil profiles with soil microbial biomass carbon (n = 289).

This database is compiled to examine the vertical distribution of soil microbial biomass globally [1]. Detailed information of variables in the dataset is listed in Global_vertical_SMBC_dataset_2.txt. References listed in Global_vertical_SMBC_dataset_2.txt can be found in Global_vertical_SMBC_dataset_ref.docx. Variables in this dataset include ecosystem, latitude (N, °), longitude (E, °), mean annual temperature (MAT, °C), MAT zone, mean annual precipitation (MAP, mm), MAP zone, soil texture, soil texture type, soil profile id, soil depth (cm), soil organic carbon (SOC, g kg^−1^), soil microbial biomass carbon (SMBC, mg kg^−1^), standard errors and sample sizes of SOC and SMBC, soil microbial quotient (SMQ, the ratio of SMBC to SOC, %), soil total nitrogen (STN, g kg^−1^), C/N ratio, soil pH, clay content (%), and reference. The data of MAT, MAP, soil depth, pH, clay content, SOC, SMBC, and SMQ are from 289 soil profiles (Fig. 1), while the data of STN and soil C/N ratio are from 153 soil profiles. To assess the effects of soil texture on the vertical distributions of SMBC and SMQ, soil textures were grouped into sandy soil (sand and loamy sand), loamy soil (sandy loam, sandy clay loam, clay loam, silt loam, loam, and silty clay loam), and clay soil (clay and silty clay).

## 3 Experimental design, materials and methods

The variables in the dataset are compiled from literature by searching the Web of Science and the China National Knowledge Infrastructure (1970–2019). The keywords used to search relevant publications are microbial biomass, soil depth, soil profile, deep soil(s), subsoil(s), and vertical. All publications included in the dataset meet the following criteria: 1) a soil profile has at least three mineral soil layers. The litter or organic soil layer was excluded, because soil properties of organic layers vary greatly across sites and most studies focus on mineral soils. 2) SMBC is measured using the chloroform-fumigation extraction method. We only included studies that use the chloroform-fumigation extraction method, because it was the most commonly used method to measure SMBC and the data were thus more available compared to the other methods, such as phospholipid fatty acid [2], deoxyribonucleic acid extraction [3], and substrate induced respiration [4].

## 4 Ethics statement

No conflict of interest exists in this submission. The authors declare that the work described in this paper is original and not under consideration for publication elsewhere, in whole or in part. Its publication is approved by all the authors listed.

## Declaration of Competing Interest

The authors declare that they have no known competing financial interests or personal relationships which have, or could be perceived to have, influenced the work reported in this article.

## Appendix Supplementary materials

Image, application 1
